# Hispolon Induces Apoptosis, Suppresses Migration and Invasion of Glioblastoma Cells and Inhibits GBM Xenograft Tumor Growth In Vivo

**DOI:** 10.3390/molecules26154497

**Published:** 2021-07-26

**Authors:** Kuan-Fu Liao, Tsung-Lang Chiu, Shu-Fang Chang, Mei-Jen Wang, Sheng-Chun Chiu

**Affiliations:** 1 Department of Internal Medicine, Taichung Tzu Chi Hospital, Buddhist Tzu Chi Medical Foundation, Taichung 427, Taiwan; kuanfuliaog@gmail.com; 2 Division of Neuro-Oncology, Neuro-Medical Scientific Center, Hualien Tzu Chi Hospital, Buddhist Tzu Chi Medical Foundation, Hualien 970, Taiwan; poluschiou@gmail.com; 3 Department of Research, Taichung Tzu Chi Hospital, Buddhist Tzu Chi Medical Foundation, Taichung 427, Taiwan; fantac10@gmail.com; 4 Department of Medical Research, Hualien Tzu Chi Hospital, Buddhist Tzu Chi Medical Foundation, Hualien 970, Taiwan; 5 Department of Laboratory Medicine, Taichung Tzu Chi Hospital, Buddhist Tzu Chi Medical Foundation, Taichung 427, Taiwan; 6 General Education Center, Tzu Chi University of Science and Technology, Hualien 970, Taiwan

**Keywords:** hispolon, glioblastoma, apoptosis, cell cycle G2/M arrest, *Phellinus linteus*

## Abstract

Hispolon, a polyphenol compound isolated from *Phellinus linteus*, has been reported to exhibit antioxidant, antiproliferative, and antitumor activities. This study aimed to explore the antitumor effects of hispolon on glioblastoma multiforme (GBM) cells in vitro and in vivo. The results revealed that hispolon significantly inhibited GBM cell proliferation and induced apoptosis through caspase-9 and caspase-3 activation and PARP cleavage. Hispolon also induced cell cycle G2/M phase arrest in GBM cells, as supported by flow cytometry analysis and confirmed by a decrease in cyclin B1, cdc2, and cdc25c protein expressions in a dose- and time-dependent manner. Furthermore, hispolon suppressed the migration and invasion of GBM cells by modulating epithelial–mesenchymal transition (EMT) markers via wound healing, transwell assays, and real-time PCR. Moreover, hispolon significantly reduced tumor growth in DBTRG xenograft mice and activated caspase-3 in hispolon-treated tumors. Thus, our findings revealed that hispolon is a potential candidate for the treatment of GBM.

## 1. Introduction

Glioblastoma multiforme (GBM) is the most common and malignant primary brain tumor in adults, with a median survival rate of only 12 months [1,2]. Standard treatments include surgical resection, radiotherapy, and the administration of chemotherapy drugs such as the DNA alkylating agent temozolomide (TMZ). However, this combination of regimens has only improved the median overall survival of patients from 12.1 to 14.6 months [3]. The effectiveness of current therapeutic strategies for GBM is believed to be reduced by several obstacles, including drug resistance, the invasiveness of GBM, and the presence of blood–brain barrier, which limits drug delivery [4,5,6]. Thus, the development of potential complementary substances to improve GBM treatment is necessary.

*Phellinus linteus* (PL), a well-known medicinal mushroom called Sanghwang in Taiwan, has been widely used as a traditional herbal medicine in Asian countries to treat inflammation, gastroenteric disorders, lymphatic diseases, and various malignancies such as lung and breast cancers [7,8,9,10,11]. PL contains various bioactive components, including polysaccharides, proteoglycans, hispidin, and hispolon [12,13,14]. Hispolon, a polyphenolic compound, was firstly isolated from *Inonotus hispidus* in 1996 and from various species of Phellinus geneus such as *Phellinus igniarius*, *Phellinus merrillii*, and PL [15,16,17]. It has been shown to exhibit antioxidant [18], anti-inflammatory [19], and anticancer properties [20,21,22]. Several natural substances, including hispolon, showed a synergistic effect with TMZ in the growth inhibition of GBM cells [23]. Although a recent study reported that hispolon has an antiproliferative effect in glioblastoma U87MG cells [24], the anticancer and antimetastatic effects of hispolon on human glioblastoma have not been well studied. Thus, in this study, the antitumor effects of hispolon on the DBTRG and C6 GBM cell lines and its underlying mechanisms in vitro and in vivo were investigated.

## 2. Results

### 2.1. Effects of Hispolon on the Viability of GBM Cells

**Figure 1:**
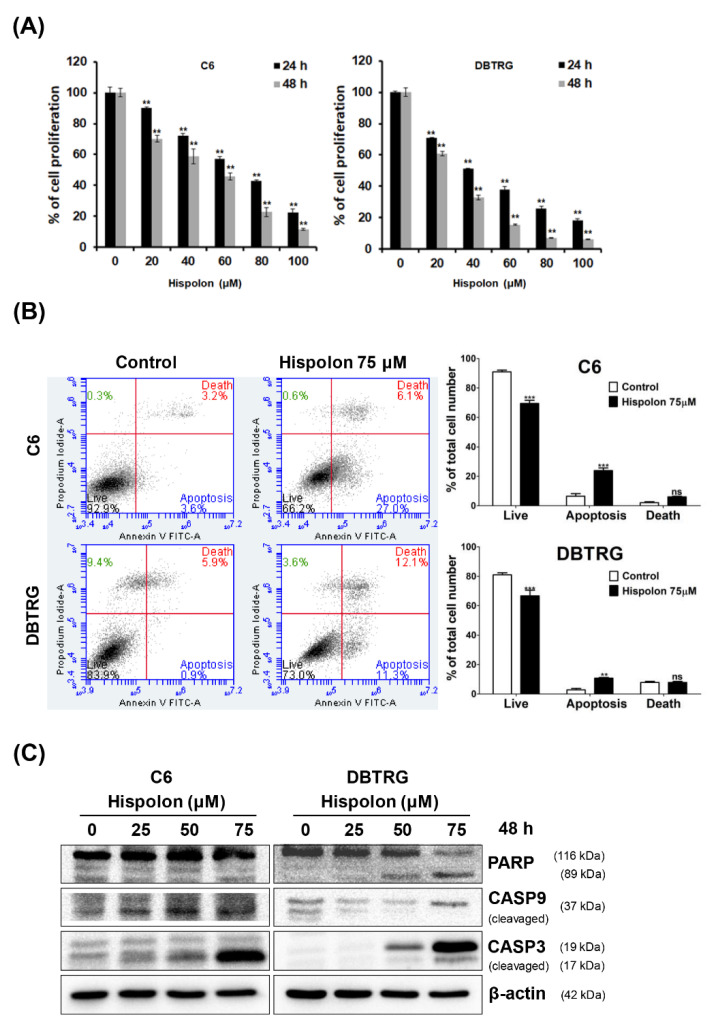
Hispolon inhibited cell proliferation and induced cell apoptosis in GBM cells. (**A**) GBM cells were treated with different concentrations of hispolon (0–100 μM) for 24 or 48 h, and the cell viability was detected through MTT assay. (**B**) GBM cells were treated with 75 μM hispolon for 24 h and then subjected to annexin V-FITC staining followed by flow cytometry analysis. (**C**) GBM cells were treated with different concentrations of hispolon (0–75 μM) for 48 h and subjected to Western blot analysis with antibodies against PARP, cleaved caspase-3, and cleaved caspase-9. Data are presented as mean ± SD. ** *p* < 0.01 versus vehicle and *** *p* < 0.001 versus vehicle, ns: not significant.

To determine the cytotoxic effect of hispolon in GBM cell lines, cells were treated with increasing concentrations of hispolon (0–100 μM) for 24 or 48 h and analyzed through MTT assay (Figure 1A). The results showed that hispolon significantly reduced the viability of C6 and DBTRG cells in a dose- and time-dependent manner. The IC_50_ values of hispolon were 68.1 and 51.7 μM on C6 cells and 55.7 and 46.6 μM on DBTRG cells for 24 and 48 h, respectively. In addition, DBTRG cells were more sensitive to hispolon-induced cytotoxicity than C6 cells.

### 2.2. Hispolon Induces Apoptosis and Caspase Activation in GBM Cells

Annexin V-FITC staining was performed on hispolon-treated C6 and DBTRG cells to elucidate the effects of hispolon on apoptosis in GBM cells. Annexin V-positive and PI-negative cells were considered early apoptotic cells. The apoptosis ratio after hispolon (75 μM) treatment was increased to 27% (C6) and 11.3% (DBTRG) compared to that in the control group (C6, 3.6%; DBTRG, 0.9%; Figure 1B). To further explore the mechanism of hispolon-induced apoptosis, cells were treated with various concentrations of hispolon (0–75 μM), and the protein expression levels of PARP, cleaved caspase-3, and cleaved caspase-9 were analyzed using Western blotting (Figure 1C). These results indicate that hispolon-induced apoptosis occurs via the intrinsic mitochondrial pathway in GBM cells.

### 2.3. Hispolon Induces Cell Cycle G2/M Arrest on GBM Cells

**Figure 2:**
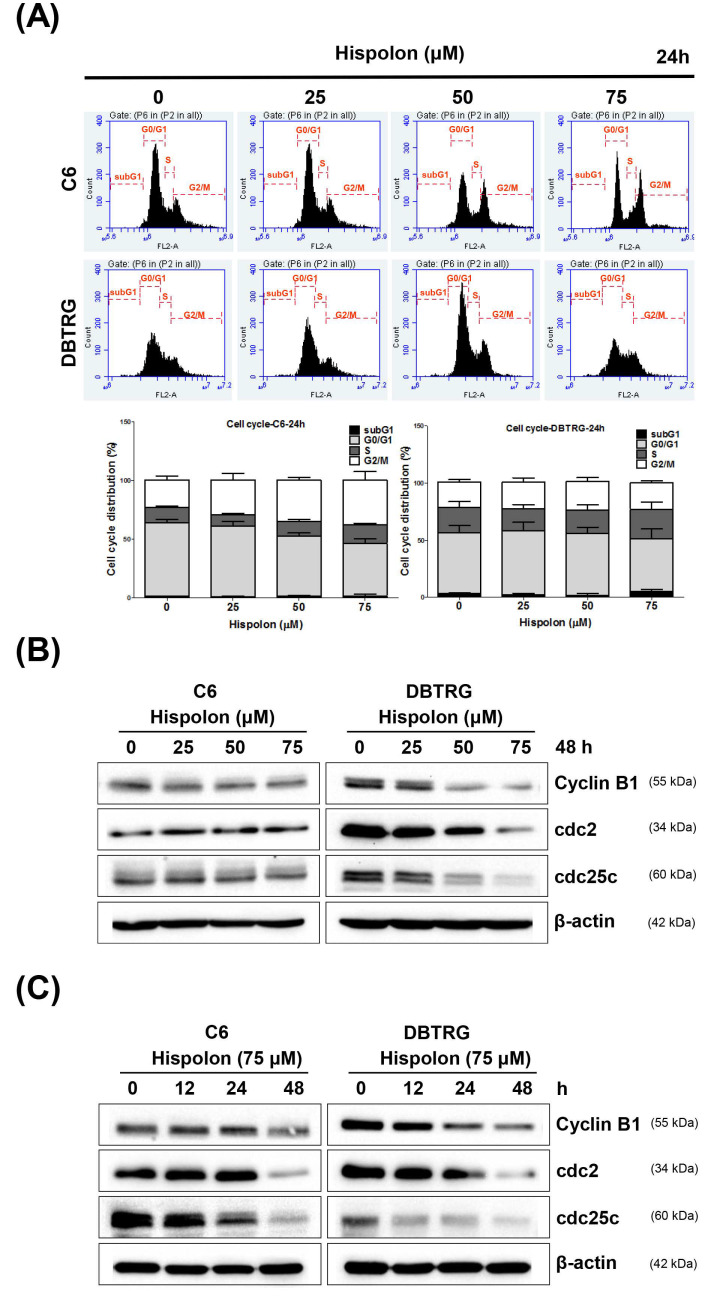
Hispolon-induced cell cycle G2/M arrest in GBM cells. (**A**) GBM cells were incubated with various concentrations of hispolon for 24 h and then subjected to PI staining followed by flow cytometry analysis. (**B**) GBM cells were treated with an increasing concentration of hispolon for 48 h, and the cell cycle regulatory proteins cyclin B1, cdc2, and cdc25c were analyzed using Western blotting. (**C**) GBM cells were treated with 75 μM hispolon for 12, 24, and 48 h, and the cell cycle regulatory proteins cyclin B1, cdc2, and cdc25c were analyzed using Western blotting.

To investigate the antiproliferative activity of hispolon on GBM cells, the cells were treated with various concentrations of hispolon (0–75 μM) for 24 h. The cell cycle profile was analyzed using DNA content measurement with propidium iodide (PI) staining and analyzed using flow cytometry (Figure 2A). Hispolon induced an increased proportion of cells in the G2/M phase (C6, 37.9%; DBTRG, 23.2%) after treatment with 75 μM hispolon, compared with the control group (C6, 23.3%; DBTRG, 21.8%). To assess the mechanism of hispolon-induced cell cycle arrest, we examined the expression levels of cell cycle G2/M phase regulatory proteins in hispolon-treated GBM cells. Hispolon treatment decreased the expression of cyclin B1, cdc2, and cdc25c in a dose- and time-dependent manner in both GBM cells (Figure 2B,C). These results suggest that hispolon induced cell cycle G2/M arrest by modulating the expression of cell cycle regulatory proteins in GBM cells.

### 2.4. Hispolon Inhibits Cell Migration and Invasion in GBM Cells

**Figure 3:**
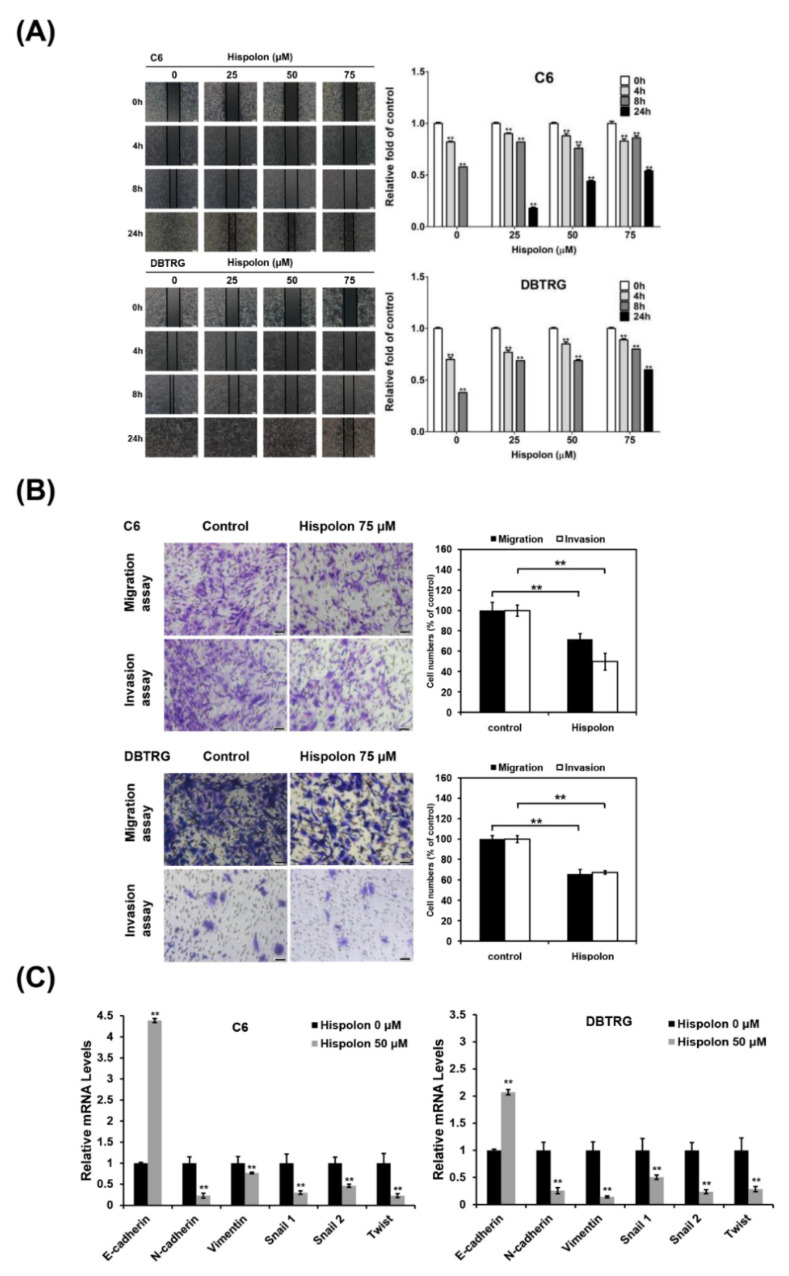
The antimetastasis effect of hispolon in GBM cells. (**A**) Hispolon inhibits C6 and DBTRG cell migration in a dose-dependent manner analyzed through the wound-healing assay. Images were captured using a microscope with 100× magnification; scale bar, 100 μm. (**B**) GBM cells were pretreated with 75 μM hispolon for 24 h and seeded onto the transwell hanging insert coating with (invasion) or without (migration) Matrigel for 24 h. Images were captured in 200× magnification and quantified by enumerating the stained cells that migrated onto the bottom of the hanging insert; scale bar, 50 μm. (**C**) GBM cells were treated with 50 μM hispolon for 24 h followed by qRT-PCR analysis. The mRNA expression levels of E-cadherin, N-cadherin, vimentin, Snail 1, Snail 2, and Twist were determined. Data are presented as means ± SD from three different experiments. ** *p* < 0.01 versus vehicle.

To explore the effect of hispolon on the migratory and invasive abilities of GBM cells, cells were treated with hispolon followed by wound-healing and transwell assay analysis. The results of the wound-healing assay revealed that hispolon (75 μM) decreased the migration of both GBM cells after 24 h of treatment compared to the control group (C6, 56%; DBTRG, 57%; Figure 3A). In addition, the transwell assay showed that hispolon significantly inhibited the migration (C6, 71.9%; DBTRG, 66%) and invasion (C6, 50%; DBTRG, 67.6%) of GBM cells compared to the control group (Figure 3B). Epithelial–mesenchymal transition (EMT) plays an important role in tumor metastasis and contributes to GBM-related mortality. Thus, we examined the expression of EMT-related genes in hispolon-treated GBM cells through qRT-PCR. The results showed that hispolon treatment upregulated the expression of the epithelial marker E-cadherin and downregulated the mesenchymal markers, including N-cadherin, in both GBM cells (Figure 3C). Together, these results indicated that hispolon inhibited the migration and invasion of GBM cells in vitro, possibly through the regulation of EMT.

### 2.5. Hispolon Suppresses Tumor Growth in DBTRG Xenograft Mice

**Figure 4:**
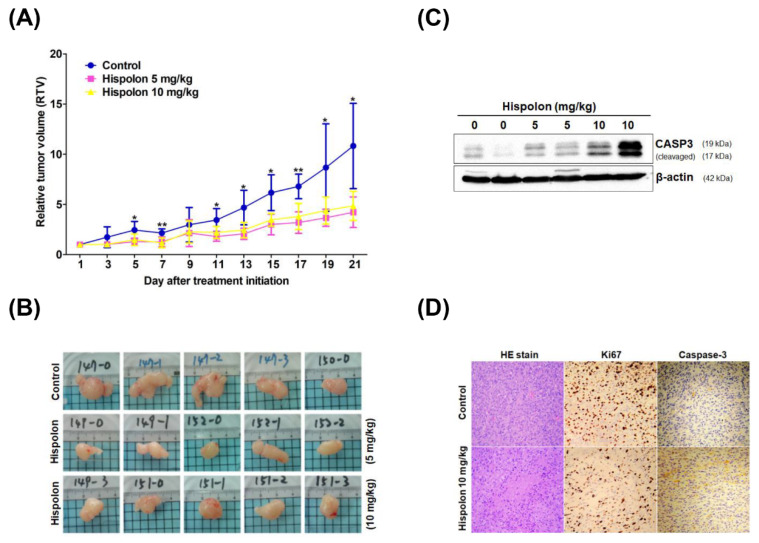
Hispolon inhibits tumor growth on DBTRG xenograft mice model. (**A**) NOD-SCID mice were injected with 1 × 10^6^ DBTRG cells into the right dorsal subcutaneous tissue. Subcutaneous injections of hispolon (■, 5 mg/kg; ▲, 10 mg/kg) were initiated when the tumor reached an average size of 150 mm^3^. The relative tumor volumes were evaluated through analysis of variance with the Games–Howell test used as a post hoc test. * *p* < 0.05 versus vehicle and ** *p* < 0.01 versus vehicle. (**B**) Tumors in the control and hispolon-treatment groups were removed and recorded on day 21. (**C**) Hispolon induced the expression of cleaved caspase-3 in tumor tissues compared with control groups, as seen in western blot analysis. (**D**) Tumor tissue sections with HE, ki-67, and caspase-3 immunohistochemical staining. The ki-67- and caspase-3-positive cells were stained brown. The expression of ki-67 in tumor tissue was downregulated and the expression of caspase-3 was upregulated after hispolon administration compared with that in the control groups.

To evaluate the antitumor effect of hispolon in vivo, a xenograft tumor-bearing model was established by transplanting DBTRG cells into the dorsal subcutaneous tissue of NOD-SCID mice. When the tumors reached ~150 mm^3^, the DBTRG xenograft mice were treated with hispolon (5 and 10 mg/kg) or the vehicle control every two days until the end of the experiments. As shown in Figure 4A, the relative tumor volume (RTV) in hispolon-treated mice was smaller than that in the vehicle control on day 21 (5 mg/kg, 61%, *p* = 0.011; 10 mg/kg, 55%, *p* = 0.015). Tumors from each group were removed and recorded (Figure 4B). To examine whether hispolon exhibits antitumor effects via apoptosis induction in vivo, the expression levels of cleaved caspase-3 in each group were analyzed using western blotting (Figure 4C). The activation of caspase-3 in hispolon-treated tumors was greater than that in the vehicle controls, especially in the 10 mg/kg hispolon-treated group. Hispolon inhibited GBM cell proliferation in vivo upon HE and ki-67 staining (Figure 4D). In addition, upregulation of caspase-3 expression in hispolon-treated tumors was also revealed through immunohistochemical staining (Figure 4D). Together, these results indicate that the induction of apoptosis is involved in hispolon-induced GBM cell death in vivo. 

## 3. Discussion

The present study investigated the antitumor effects of hispolon on GBM and its underlying mechanisms. We demonstrated the antiproliferative activity of hispolon in two GBM cell lines in vitro. The IC_50_ values of hispolon on these GBM cells (46.6–57.7 μM) were higher than those in other tumor cells such as prostate cancer (28–31 μM), bladder cancer (20–40 μM), and acute myeloid leukemia (6.5–25 μM). In vivo results showed that subcutaneous administration of hispolon at a dose of 5 or 10 mg/kg caused a reduction in the size of DBTRG xenograft tumor masses. The increased expression of cleaved caspase-3 implies that hispolon exerts antitumor activity via apoptosis induction. This is the first demonstration of the antitumor effect of hispolon on GBM in vitro and in vivo.

Several reports have indicated that hispolon blocks cell cycle progression at the G0/G1 [25,26] or G2/M [16,27] phase, depending on the cell context. In the present study, hispolon inhibited cell cycle progression and induced cell cycle arrest at the G2/M phase in C6 cells. However, the hispolon-treated DBTRG cells accumulating more cells in the S phase than in G2/M phase was noted. This discrepancy of cell cycle regulation might be due to the differences of the used cell lines. Treatment with hispolon for 24 h caused an increase in the number of cells in the G2/M phase in a dose-dependent manner in GBM cells, as seen in FACS analysis (Figure 2A). Similarly, hispolon induces cell cycle G2/M arrest by downregulating CDK4 and upregulating p21 and p53 in GBM U87MG cells, as reported by Arcella et al. [24]. To further explore the mechanism of hispolon-induced cell cycle arrest, the expression of cell cycle G2/M regulatory proteins was analyzed using Western blotting. Accordingly, hispolon inhibited the expression of cyclin B1, cdc2, and cdc25c in a time- and dose-dependent manner (Figure 2B,C). Our results indicated that hispolon-mediated cell cycle G2/M arrest might be correlated with the downregulation of cyclin B1, cdc2, and cdc25c proteins. 

Recent studies have indicated that hispolon exhibits antitumor activity by inducing apoptosis in nasopharyngeal carcinoma (NPC), leukemia, hepatocellular carcinoma, and gastric cancer [20,28,29,30]. Hispolon also induced ERK1/2 phosphorylation, stimulating the activation of caspase-3 and the cleavage of PARP in GBM U87MG cells [24]. Consistently, our results suggested that hispolon induced mitochondria-mediated apoptosis in GBM cells via the activation of caspase-9 and caspase-3 and the cleavage of PARP. In addition, hispolon induced caspase-3 activation and suppressed tumor growth in DBTRG-xenograft mice in a dose-dependent manner.

Metastasis is the main feature that contributes to GBM spread and mortality [31]. EMT is an important process that enables cells to lose their adhesive properties and increase motility to metastasize to distant regions. The most common characteristic of EMT is the loss of E-cadherin expression and the acquisition of N-cadherin expression. EMT is regulated by several transcriptional factors, such as Snail1, Snail2 (Slug), and Twist [32]. Our results showed that hispolon can significantly inhibit the migration and invasion of GBM cells by wound-healing and transwell assays. In addition, hispolon treatment markedly reduced the expression of N-cadherin, vimentin, Snail1, Snail2, and Twist and induced E-cadherin expression (Figure 3). Hispolon has been reported to inhibit transforming growth factor-β (TGF-β)-induced cell migration and invasion by downregulating the expression of Snail and Twist [33]. These results suggest that hispolon could exerts antimetastatic effects by inhibiting EMT in GBM cells.

TMZ is a DNA alkylating agent that induces cell cycle G2/M phase arrest and leads to apoptosis in cancer cells. TMZ was approved by the United States Food and Drug Administration in 2005 and is now the first-line chemotherapy drug of choice for GBM therapy. Combination therapy regimens have become more effective in treating many human malignancies, including GBM. The synergistic effect of a combination of hispolon (25 μM) and TMZ (10 μM) on U87MG cells has been reported [24]. Concomitant treatment with hispolon and TMZ significantly increased the antiproliferative effect of each drug individually. The side effects can also be expected to be reduced because these drugs are used at a lower dose than those used in monotherapy. Considering the poor survival rates of patients with GBM, hispolon might be a natural adjuvant candidate to improve GBM therapy. 

## 4. Materials and Methods

### 4.1. Glioblastoma Cell Lines

The human glioblastoma cell line DBTRG and rat glioblastoma cell line C6 were purchased from the Bioresource Collection and Research Center (Hsinchu, Taiwan). Each cell line was cultured in standard medium, as recommended by the BCRC. Culture medium and fetal bovine serum were purchased from Invitrogen (Thermo Fisher Scientific, Carlsbad, CA, USA). Cell lines were authenticated annually by short tandem repeat analysis and routinely tested for mycoplasma contamination.

### 4.2. Chemicals and Reagents

Hispolon was purchased from Santa Cruz Biotechnology (Dallas, TX, USA). All other chemicals were purchased from Sigma Chemical Co. (St. Louis, MO, USA). The antibodies were purchased from Cell Signaling Technology, Inc. (Danvers, MA, USA). The Bradford protein assay kit was purchased from Bio-Rad (Hercules, CA, USA). PVDF membranes were purchased from Merck Millipore (Billerica, MA, USA). Western blot chemiluminescence reagents were purchased from Amersham Biosciences (Arlington Heights, IL, USA).

### 4.3. Cell Viability Assay

Cell viability was evaluated using the MTT assay as described [34].

### 4.4. RNA Extraction and Real-Time RT-PCR

Total RNA was extracted from GBM cells using the RNeasy Mini kit (Qiagen, Valencia, CA, USA) and reverse transcribed at 37 °C for 60 min using an Omniscript RT kit (Qiagen, Valencia, CA, USA) according to the manufacturer’s instructions. Real-time RT-PCR was performed, and the primer sequences are listed as described [35].

### 4.5. Western Blot

Western blot analysis was performed as described [34].

### 4.6. Cell Cycle Analysis

Cell cycle analysis was performed through PI staining to reveal the DNA content and analyzed through flow cytometry, as described previously [34]. Briefly, cells were collected, washed with PBS, and fixed with 70% ethanol overnight. Cells were then stained with a solution containing 20 μg/mL PI, 0.2 mg/mL RNase A, and 0.1% Triton X-100 for 30 min in the dark, followed by flow cytometry analysis.

### 4.7. Cell Migration and Invasion Assay

The wound-healing assay was performed as previously described [36]. Briefly, GBM cells were seeded in a 24-well plate with culture inserts (ibidi, Martinsried, Germany) for 24 h. The cells were washed with PBS and cultured with medium containing 0–75 μM hispolon for 24 h. Wound closure was photographed and evaluated at 0, 4, 8, and 24 h using an inverted microscope (Olympus CKX41 microscope, Melville, NY, USA).

Cell migration and invasion assay were performed as previously described [35]. Briefly, GBM cells were treated with hispolon for 24 h and then reseeded in the upper chambers with (invasion assay) or without (migration assay) Matrigel coating (2 mg/mL) in a 24-well plate for another 24 h. Culture medium containing 10% FBS was used as a chemoattractant in the lower chambers. The invaded cells were fixed with 10% formalin and stained with 0.2% crystal violet for 15 min, photographed, and counted at 200× magnification.

### 4.8. Animal Studies

Ethics Statement: The animal use protocol listed below was reviewed and approved by the Institutional Animal Care and Use Committee (IACUC), Hualien Tzu Chi Hospital, approval no.: 107-03. All procedures were performed in compliance with the standard operating procedures and the relevant guidelines of the Tzu Chi University Laboratory Animal Center (Hualien, Taiwan). Tumors were generated as described previously [34]. Briefly, 10^6^ DBTRG cells were subcutaneously implanted into the backs of NOD-SCID mice. Mice were divided randomly into control and treatment groups consisting of six mice per group after the tumor volume reached 80–150 mm^3^. Subcutaneous injections (contralateral flank to tumor site) of 5 or 10 mg/kg hispolon (treatment groups) were administered every 2 days until the end of the experiments. The tumor volume was determined by measuring the length (L) and width (W) of the tumor and calculated as TV (mm^3^) = (L × W^2^)/2. The RTV at day n versus day 0 was expressed as RTVn = TVn/TV0. On day 0, treatment was started. Xenograft tumors were harvested, photographed, fixed with 4% formalin, and embedded in paraffin for histological analysis.

### 4.9. Statistical Analysis

All data were analyzed using Student’s *t*-test for normally distributed values and the nonparametric Mann–Whitney U test for values with a nonnormal distribution, as described [35,37].

## 5. Conclusions

In summary, the current study showed for the first time that hispolon exerts antitumor activity on GBM cells in vitro and in vivo. The antiproliferative activity of hispolon can be attributed to (1) apoptosis induction via mitochondrial pathways by inducing caspase-9 and caspase-3 activation, and (2) cell cycle G2/M phase arrest induction by modulating the expression of cell cycle regulatory proteins. Hispolon also inhibits the migration and invasion of GBM cells by suppressing the EMT signals. Furthermore, hispolon significantly activated caspase-3 expression and suppressed tumor growth in DBTRG xenograft mice. Our findings revealed that hispolon may be a potential candidate as a chemotherapeutic agent for GBM therapy.

## Data Availability

Not applicable.

## Sample Availability

Samples of the compounds are not available from the authors.
